# Supplementary material for: Conformational cycle of human polyamine transporter ATP13A2
Source: Nat Commun. 2023 Apr 8;14:1978. doi: 10.1038/s41467-023-37741-0 (PMC10082790; doi:10.1038/s41467-023-37741-0)
Supplement: Supplementary file 1 — Supplementary Information [file 41467_2023_37741_MOESM1_ESM.pdf]

Supplementary Information

Conformational cycle of human polyamine transporter ATP13A2

Jianqiang Mu[1,6], Chenyang Xue[1,6], Lei Fu[2,6], Zongjun Yu[1], Minhan Nie[4], Mengqi Wu[1], Xinmeng Chen[1], Kun Liu[1], Ruiqian Bu[1], Ying Huang[1], Baisheng Yang[1], Jianming Han[1], Qianru Jiang[1], Kevin C. Chan[2], Ruhong Zhou[2], Huilin Li[4,5], Ancheng Huang[1], Yong Wang[2,3]*, Zhongmin Liu[1]*

[1]*Department of Immunology and Microbiology, School of Life Sciences, Southern University of Science and Technology, Shenzhen 518055, Guangdong, China*

[2] *Shanghai Institute for Advanced Study, Institute of Quantitative Biology, College of Life Sciences, Zhejiang University, Hangzhou 310027, China*

[3]*The Provincial International Science and Technology Cooperation Base on Engineering Biology, International Campus of Zhejiang University, Haining, 314400, China*

[4]*School of Pharmaceutical Sciences, Sun Yat-sen University, No.132 Wai Huan Dong Lu, Guangzhou Higher Education Mega Center, Guangzhou, 510006, China*

[5]*Guangdong Key Laboratory of Chiral Molecule and Drug Discovery, School of Pharmaceutical Sciences, Sun Yat-sen University, Guangzhou, Guangdong, 510006, China*

[6]*These authors contributed equally to this work*

*Correspondence and lead contact: yongwang_isb@zju.edu.cn, liuzm@sustech.edu.cn

Supplementary Fig. 1

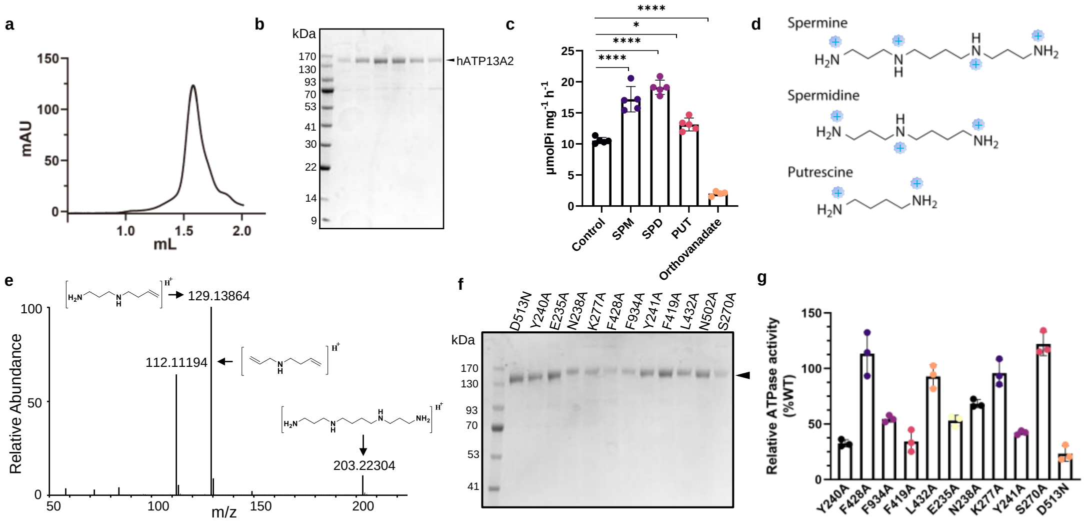

Supplementary Fig. 1 Biochemical analysis of hATP13A2.

a Size-exclusion chromatography profile of hATP13A2. **b** SDS-PAGE analysis of the hATP13A2 peak fractions in the SEC purification. A representative image of three independent experiments. **c** The ATPase activity of purified ATP13A2 was measured in different buffers containing 100 μM SPM, 100 μM SPD, 100 μM PUT, or 2 mM orthovanadate, respectively. Orthovanadate, a general P-type ATPase inhibitor. Data represent the mean ± SD of three (orthovanadate) or four (Control, SPM, SPD, and PUT) independent measurements. One-way ANOVA analysis was performed. Statistical significance compared with the wild type is shown: *p <0.05; ****p < 0.001. **d** Diagram showing the distribution of positively charged groups of SPM, SDP, and PUT. **e** LC-MS/MS analysis hATP13A2 bound endogenous polyamine. The square brackets show the product ion spectra of SPM identifying putative fragmentation pathways. **f** SDS-PAGE analysis of purified mutant hATP13A2 proteins. A representative image of three independent experiments. **g** Impacts of residue mutations at the inward-open cavity on SPM-dependent ATPase activity. The ATPase (WT or mutants) activity was measured in the presence of 100 μM SPM. D513N, the catalytically dead mutant hATP13A2, served as a positive control. Data presented as mean ± SD of three independent experiments.

Supplementary Fig. 2

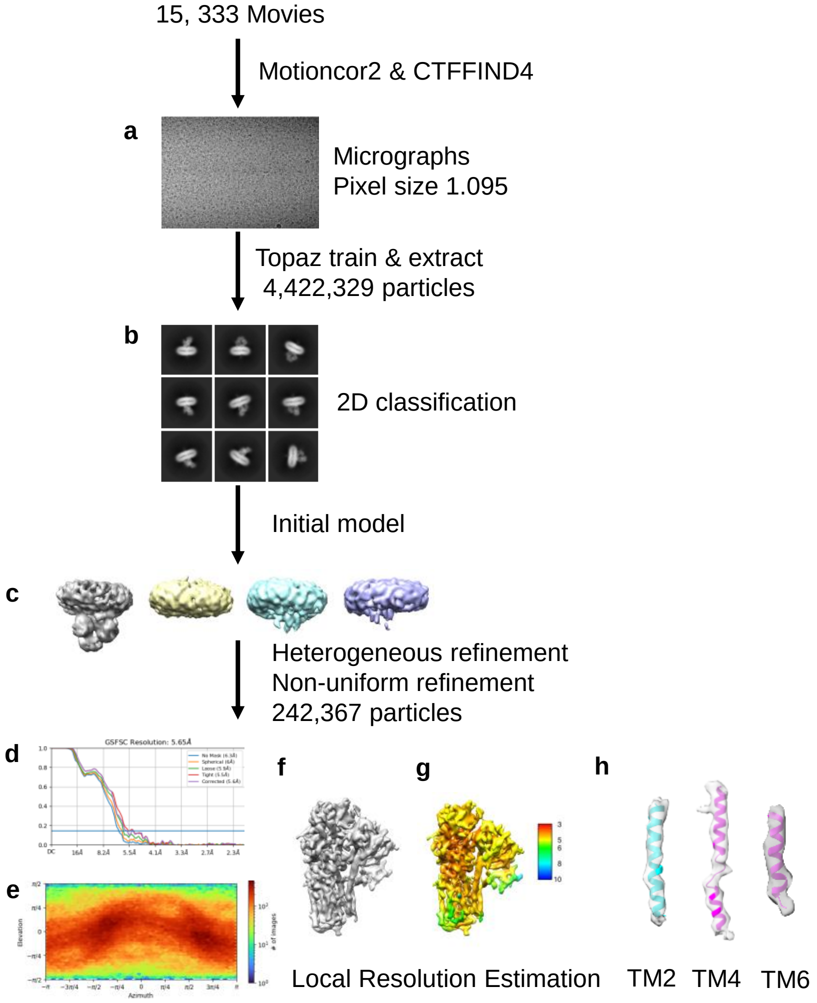

Supplementary Fig. 2 Flowchart of cryo-EM reconstruction of hATP13A2 in the E1-like state. **a-c** Representative cryo-EM image (a), 2D classification (b), and initial model (c) of the E1-like state. **d** Corrected Fourier Shell Correlation (FSC) curve of the final 3D reconstruction of the E1-like state and after non-uniform refinement at the FSC=0.143, respectively, in cryoSPARC. **e** Particle view orientation distribution of the E1-like state. **f** Cryo-EM refined map of the E1-like state. **g** Local resolution map of the final 3D reconstruction estimated using local resolution estimation. **h** Representative EM densities for TM2, TM4, and TM6 in the E1-like state.

Supplementary Fig. 3

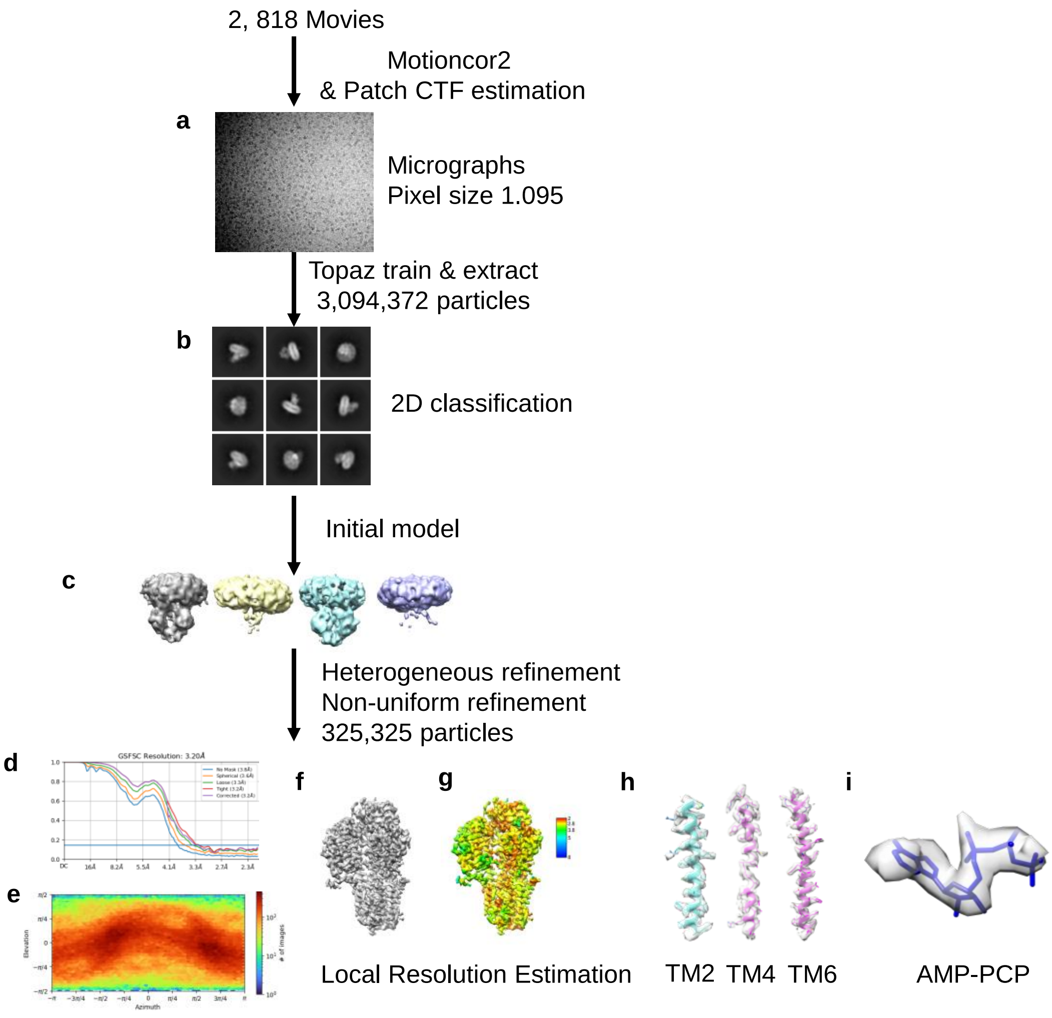

Supplementary Fig. 3 Flowchart of cryo-EM reconstruction of hATP13A2 in the E1-ATP state.

a-c Representative cryo-EM image (a), 2D classification (b), and initial model (c) of the E1-ATP state. **d** Corrected Fourier Shell Correlation (FSC) curve of the final 3D reconstruction of the E1-ATP state and after non-uniform refinement at the FSC=0.143, respectively, in cryoSPARC. **e** Particle view orientation distribution of the E1-ATP state. **f** Cryo-EM refined map of the E1-ATP state. **g** Local resolution map of the final 3D reconstruction estimated using local resolution estimation. **h** Representative EM densities for TM2, TM4, and TM6 in the E1-ATP state. **i** EM densities and atomic models of AMP-PCP.

Supplementary Fig. 4

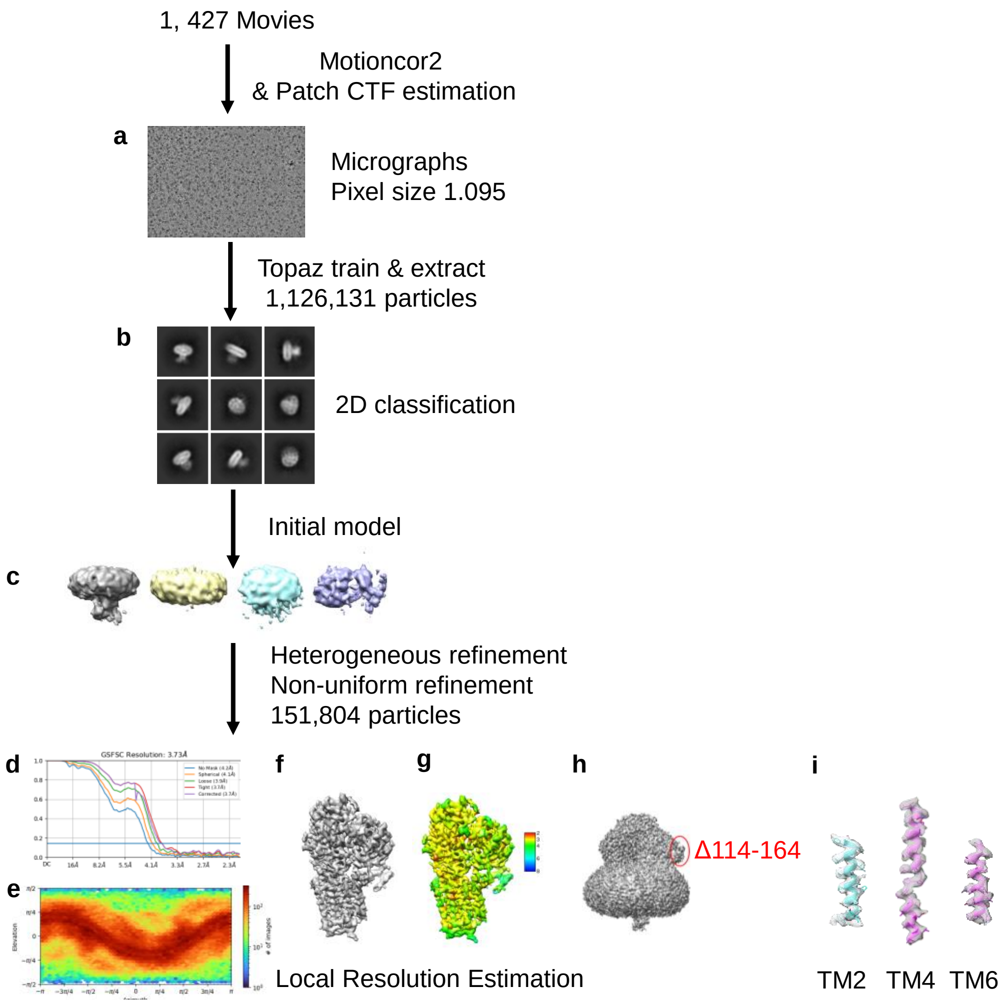

Supplementary Fig. 4 Flowchart of cryo-EM reconstruction of hATP13A2 in the E1P-ADP state.

a-c Representative cryo-EM image (a), 2D classification (b), and initial model (c) of the E1P-ADP state. **d** Corrected Fourier Shell Correlation (FSC) curve of the final 3D reconstruction of the E1P-ADP state and after non-uniform refinement at the FSC=0.143, respectively, in cryoSPARC. **e** Particle view orientation distribution of the E1P-ADP state. **f** Cryo-EM refined map of the E1P-ADP state. **g** Local resolution map of the final 3D reconstruction estimated using local resolution estimation. **h** The putative densities represent fragments ranging from E114 to R164 in NTD. **i** Representative EM densities for TM2, TM4, and TM6 in the E1P-ADP state.

Supplementary Fig. 5

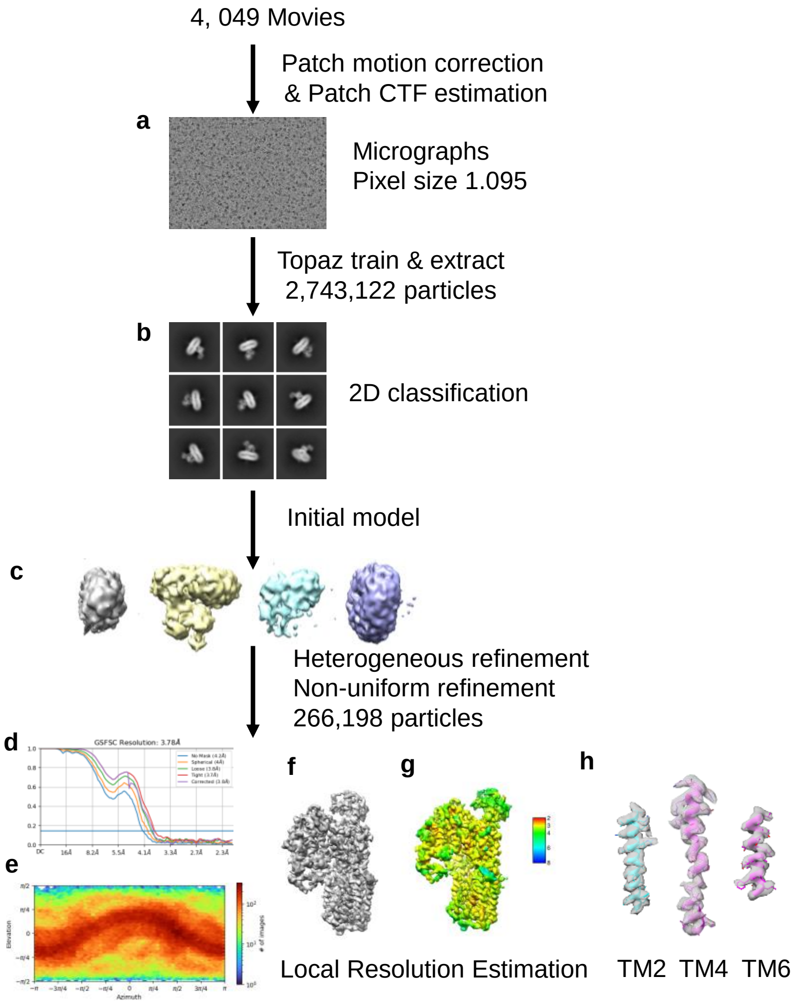

Supplementary Fig. 5 Flowchart of cryo-EM reconstruction of hATP13A2 in the nominal E1P state.

a-c Representative cryo-EM image (a), 2D classification (b), and initial model (c) of the nominal E1P state. **d** Corrected Fourier Shell Correlation (FSC) curve of the final 3D reconstruction of the nominal E1P state and after non-uniform refinement at the FSC=0.143, respectively, in cryoSPARC. **e** Particle view orientation distribution of the nominal E1P state. **f** Cryo-EM refined map of the nominal E1P state. **g** Local resolution map of the final 3D reconstruction estimated using local resolution estimation. **h** Representative EM densities for TM2, TM4, and TM6 in the nominal E1P state.

Supplementary Fig. 6

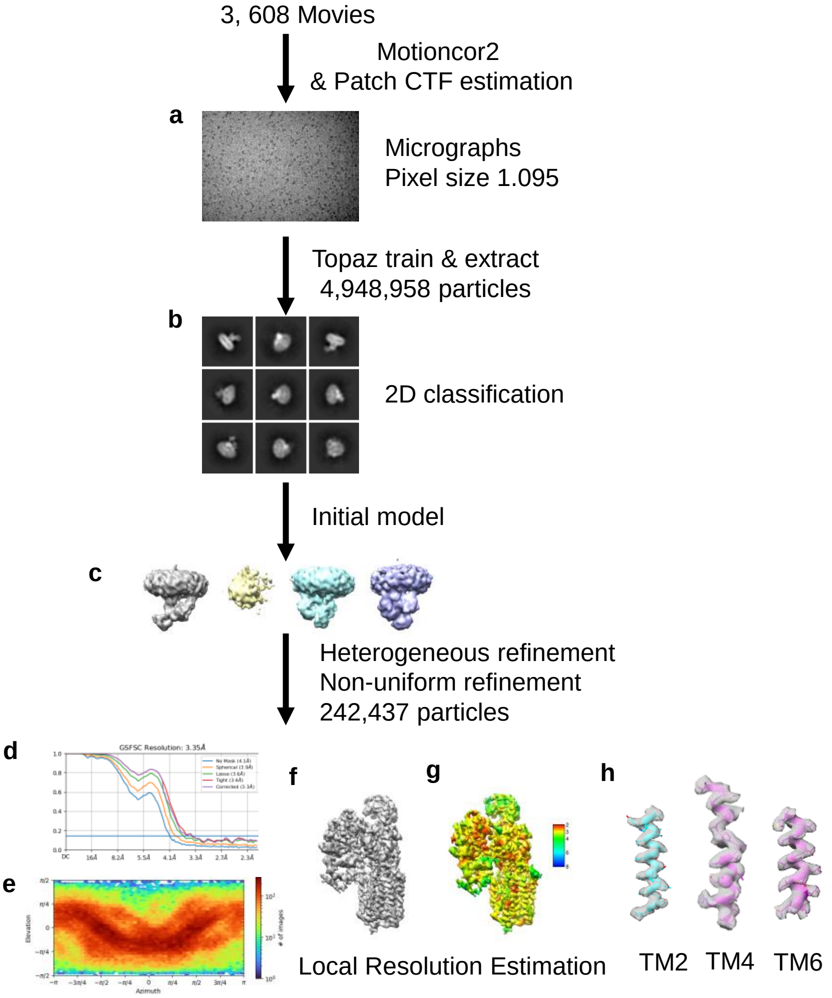

Supplementary Fig. 6 Flowchart of cryo-EM reconstruction of hATP13A2 in the E2P state. **a-c** Representative cryo-EM image (a), 2D classification (b), and initial model (c) of the E2P state. **d** Corrected Fourier Shell Correlation (FSC) curve of the final 3D reconstruction of the E2P state and after non-uniform refinement at the FSC=0.143, respectively, in cryoSPARC. **e** Particle view orientation distribution of the E2P state. **f** Cryo-EM refined map of the E2P state. **g** Local resolution map of the final 3D reconstruction estimated using local resolution estimation. **h** Representative EM densities for TM2, TM4, and TM6 in the E2P state.

Supplementary Fig. 7

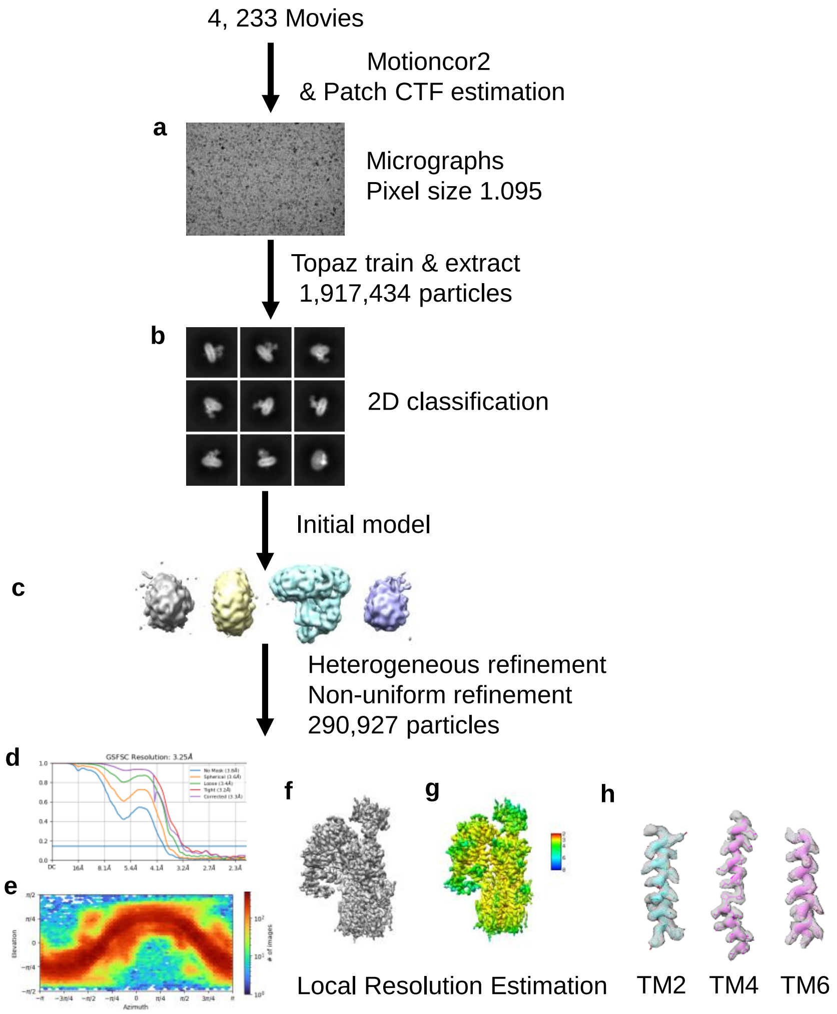

Supplementary Fig. 7 Flowchart of cryo-EM reconstruction of hATP13A2 in the E2-Pi state. **a-c** Representative cryo-EM image (a), 2D classification (b), and initial model (c) of the E2-Pi state. **d** Corrected Fourier Shell Correlation (FSC) curve of the final 3D reconstruction of the E2-Pi state and after non-uniform refinement at the FSC=0.143, respectively, in cryoSPARC. **e** Particle view orientation distribution of the E2-Pi state. **f** Cryo-EM refined map of the E2-Pi state. **g** Local resolution map of the final 3D reconstruction estimated using local resolution estimation. **h** Representative EM densities for TM2, TM4, and TM6 in theE2-Pi state.

Supplementary Fig. 8

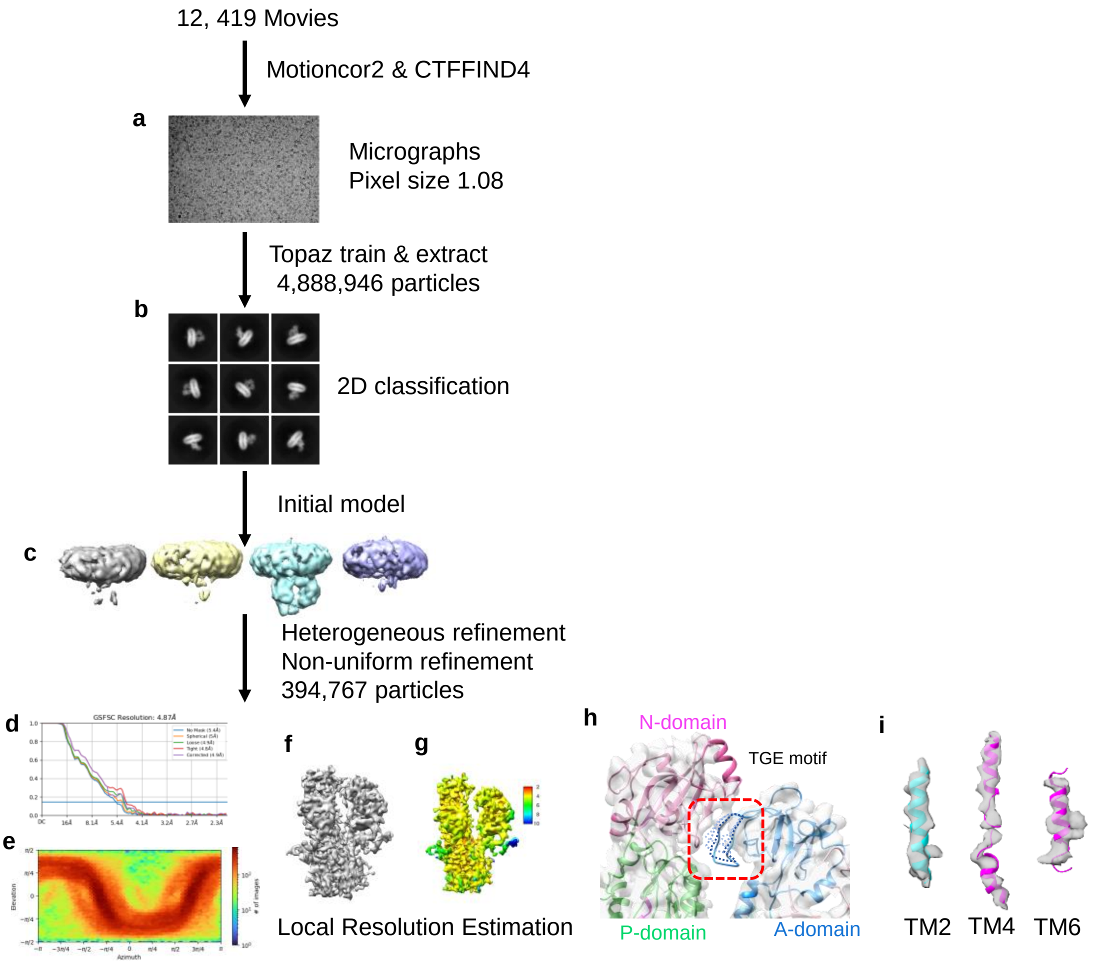

Supplementary Fig. 8 Flowchart of cryo-EM reconstruction of hATP13A2 in the putative of E2 state.

a-c Representative cryo-EM image (a), 2D classification (b), and initial model (c) of the putative E2 state. **d** Corrected Fourier Shell Correlation (FSC) curve of the final 3D reconstruction of the putative E2 state and after non-uniform refinement at the FSC=0.143, respectively, in cryoSPARC. **e** Particle view orientation distribution of the putative E2 state. **f** Cryo-EM refined map of the putative E2 state. **g** Local resolution map of the final 3D reconstruction estimated using local resolution estimation. **h** Electron density map around the TGE motif is poorly defined probably because of the structural flexibility. **i** Representative EM densities for TM2, TM4, and TM6 in the putative of E2 state.

Supplementary Fig. 9

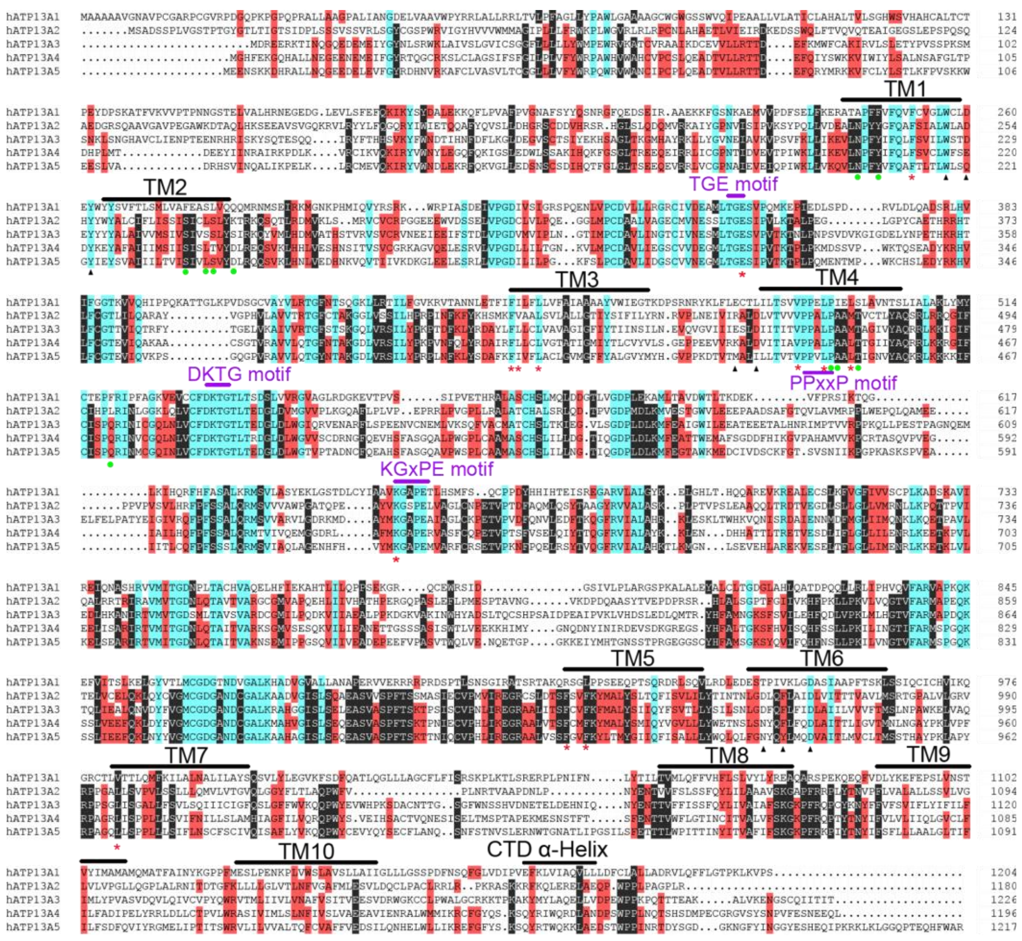

Supplementary Fig. 9 Multiple sequence alignment of five human P5B-type ATPases. Amino acids are colored according to the conservation (Cyan represents the most conservative, followed by black, then the weakest is red). Uniprot numbers are as follows: hATP13A1 (Uniprot: Q9HD20), hATP13A2 (Uniprot: Q9NQ11), hATP13A3 (Uniprot: Q9H7F0), hATP13A4 (Uniprot: Q4VNC1), and hATP13A5 (Uniprot: Q4VNC0). The transmembrane helices of hATP13A2 are indicated by the black line. The conserved KGxPE motif, DGET motif, TGE motif, and PPxxP motif are indicated by purple line. $Site_1$, $Site_2$ and the release site were represented black triangle, red asterisk and green solid circle, respectively.

Supplementary Fig. 10

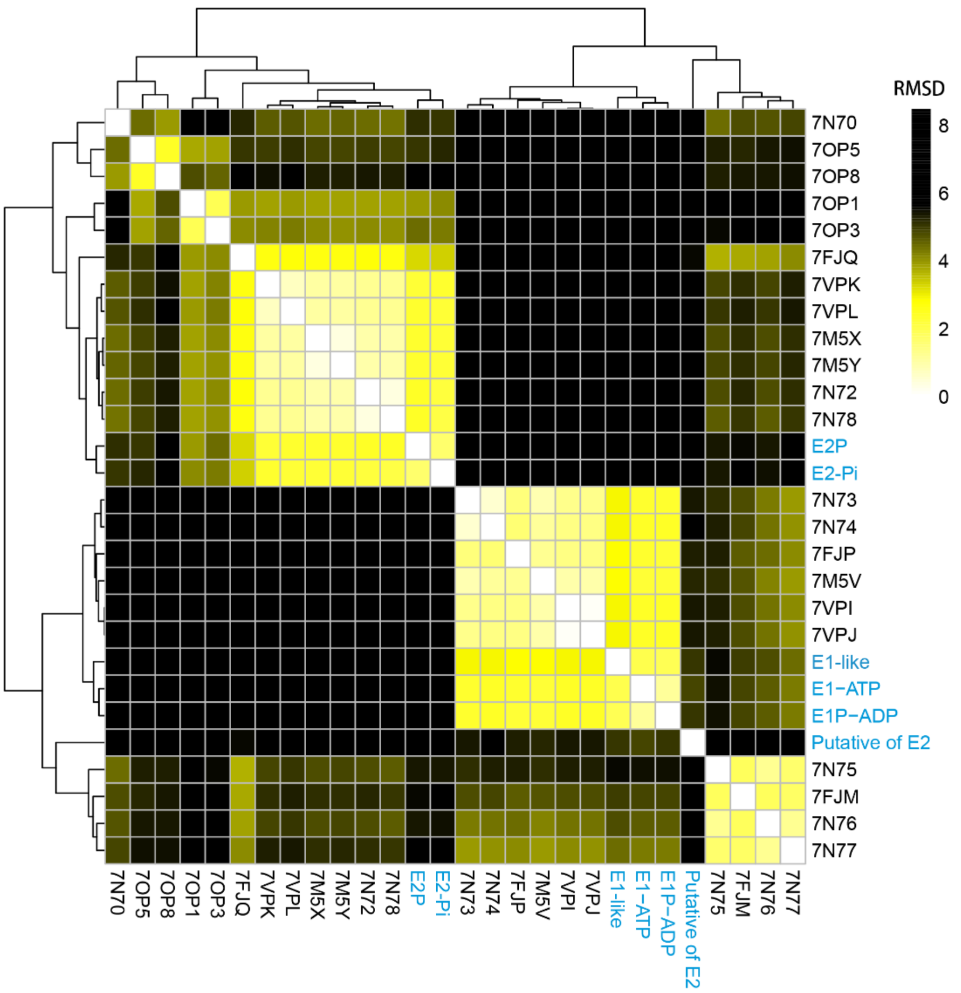

Supplementary Fig. 10 Structural comparison of our structures and recently solved structures of ATP13A2 and homologous proteins in different intermediate states. The structural information is summarized in Supplementary Table 3**.**

Supplementary Fig. 11

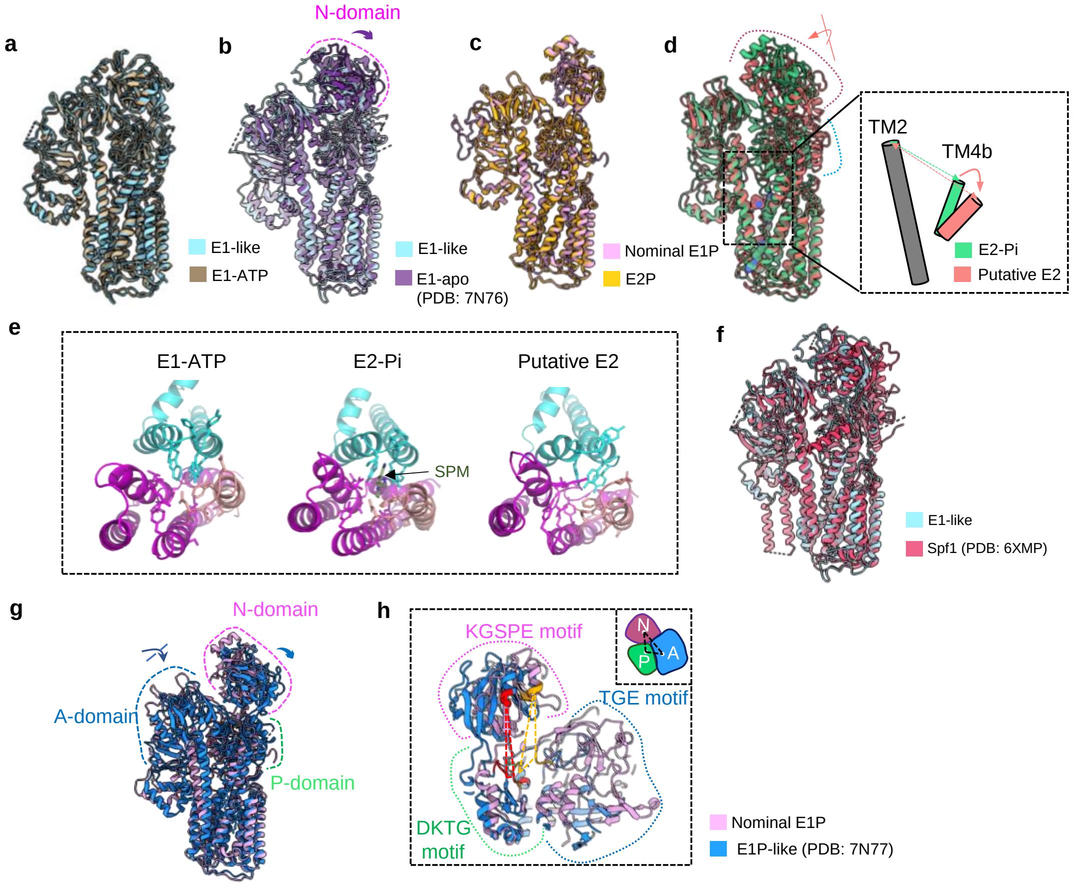

Supplementary Fig. 11 Comparison analysis of hATP13A2 structures.

a, b The structure of hATP13A2 in the E1-like state (cyan) is compared with that of the E1-ATP state (brown) (a), and the D458N/D962N mutant in the E1-apo state (PDB: 7N76, purple) (b), respectively. Arrows are used to indicate the direction of movement. **c, g** The structure of hATP13A2 in the AlF_4^- bound nominal E1P state (pink) is compared with that of the BeF_3^- stabilized E2P state (yellow) (c), and the structure of D458N/D962N mutant in the AlF_4^-bound E1P-like state (PDB:7N77, blue) (g), respectively. **d** Structure of hATP13A2 in the E2-Pi state (salmon) is compared with that of the putative E2 state (green). Zoomed-in view showing conformational changes in TM2 and TM4b helices upon E2-Pi to putative E2 transition. **e** Comparison of transmembrane regions of polyamine entry sites in E1-ATP, E2P and putative E2 state, respectively. **f** The structure of hATP13A2 in the E1-like state (cyan) is compared with the structure of the apo form of Spf1 (PDB: 6XMP, red) (f)**. h** As in g, structural changes of the A, N, and P domains are indicated by the distances of the KGSPE motif, DKTG motif, and TGE motif, respectively. The conserved motif of cytoplasmic domain in the nominal E1P state is colored in red, and the 7N77 is colored in yellow.

Supplementary Fig. 12

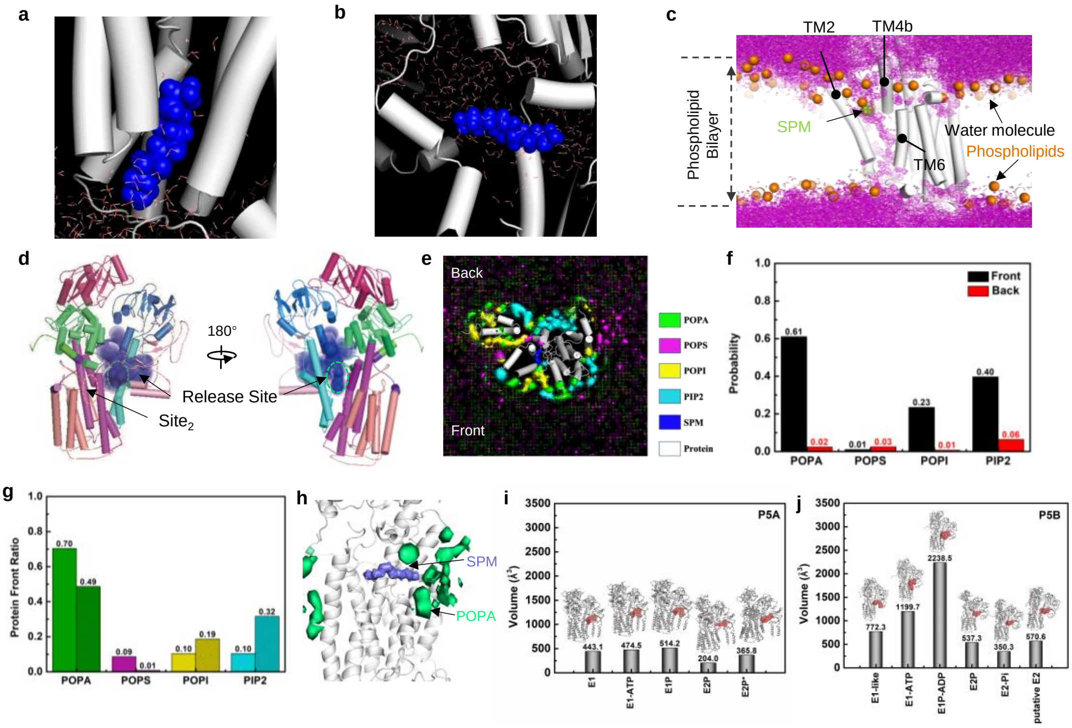

Supplementary Fig. 12 Analysis of the interaction between SPM and lipid, and hydration of the binding sites.

a, b Hydration of $Site_1$ (a) and the release site (b) observed from the all-atom MD simulations of ATP13A2 in E2P state. **c** The averaged density map of water obtained from the all-atom MD simulation of ATP13A2 in the putative of E2 state is shown as magenta meshes. **d** Tunnel analysis of the cytosolic cavity of hATP13A2 putative of E2 state. **e** The averaged lipid density map obtained from ten trajectories in CG MD simulation. **f** The probability of lipids bound on the front and back sides around the cytosolic cavity revealed that the front side has strong lipid binding in contrast to the back side. **g** Lipid selectivity is revealed by the lipid-bound probability on the front side of hATP13A2 around the cytosolic cavity. The solid-colored histograms (left) represent the reference probabilities of the four negatively charged phospholipids and the black slashed ones (right) are the simulated probabilities of the lipids identified around the cavity. **h** The average density map of POPA obtained from CG MD simulations. **i, j** The volume analysis of the cytosolic cavities of intermediate states for P5A (i) and P5B (j).

Supplementary Fig. 13

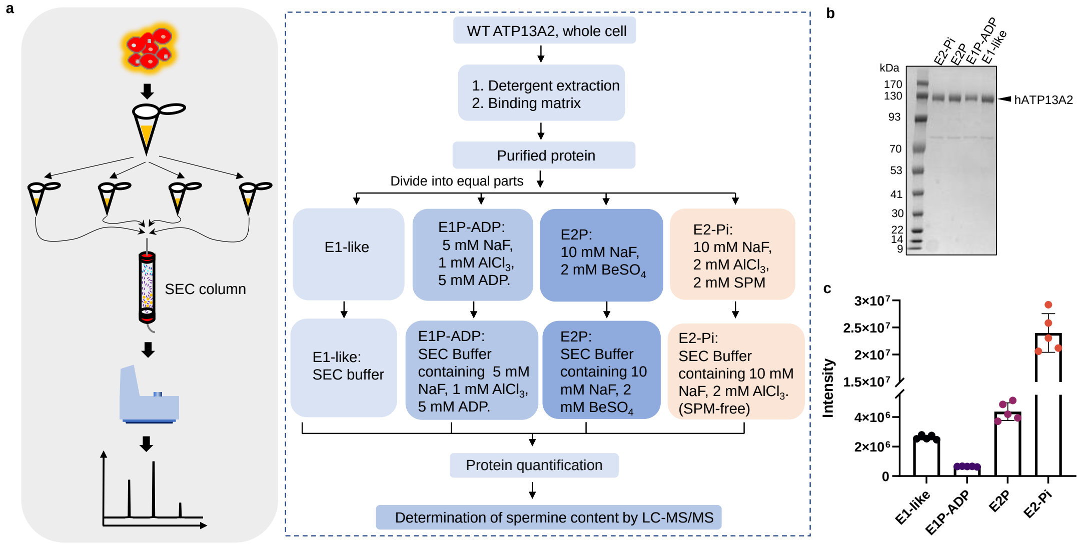

Supplementary Fig. 13 Quantification of the SPM captured by hATP13A2 in different intermediate states by LC-MS/MS.

a A schematic diagram of the determination of hATP13A2 substrate content in different intermediate transport states by LC-MS/MS (left) and a flowchart summarizing the process of quantifying polyamine in different states (right). **b** SDS-PAGE analysis of hATP13A2 variants. **c** Bar graph featuring the abundance values of SPM determined by mass spectrometry. The graph is performed as the means ± SD of five independent experiments using GraphPad Prism version 9.0.0. statistical software.

Supplementary Fig. 14

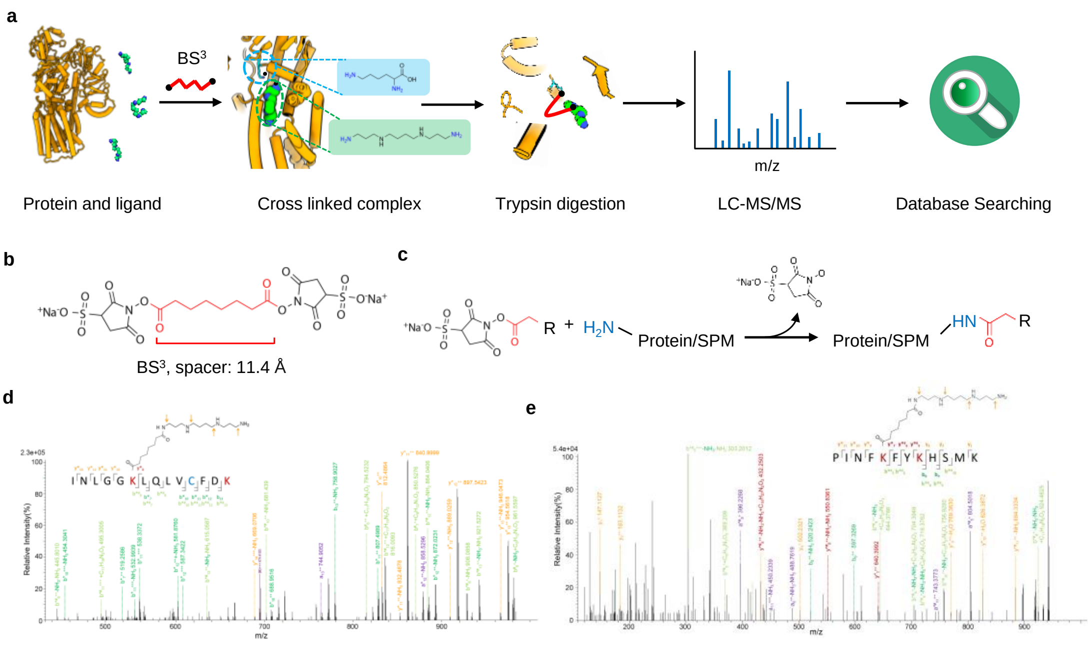

Supplementary Fig. 14 Probing potential binding sites for SPM in hATP3A2 by XL-MS. **a** XL-MS analysis workflow for identifying hATP13A2 cross-linked SPM. **b** Chemical structure of BS^3 cross-linking reagent. Spacer arm 11.4 **Å**. This cross-linker reacts with primary amines (lysine side chain, protein N-terminus). **c** Reaction of a cross-linker with a primary amine. The leaving group is part of the cross-linker is substituted with the primary amine to form a covalent bond between the spacer and the amine. R can stand for either the rest of the cross-linker or another protein or SPM once the cross-linker has already reacted on its other end. **d, e** Annotated MS2 spectrum of the protein hATP13A2 (Q9NQ11) peptide INLGGKLQLVCFDK (d) and peptide PINFKFYKHSMK (e). Matched ions are labeled in the spectrum and indicate that protein is modified by SPM on K506 (d) and K420 (e), respectively. The peptide fragments randomly on each amide bond, resulting in carboxy-terminal y ions or amino-terminal b ions.

Supplementary Fig. 15

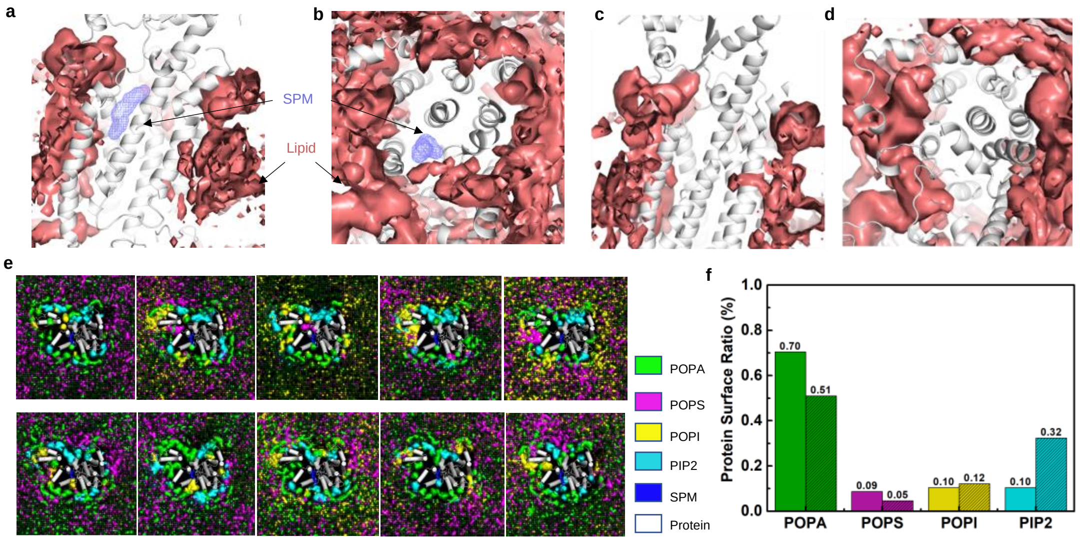

Supplementary Fig. 15 Lipid binding to hATP13A2 in the E2-Pi and putative of E2 state obtained from CG MD simulations.

a, b The bound lipids around the SPM substrates in Site$_2$ from a left sectional view (a) and a top view (b) in the E2-Pi state, respectively. **c, d** A left sectional view (c) and a top view (d) of average lipid density maps obtained from coarse-grained MD simulations of the E2-Pi state without SPM, respectively. **e** Average lipid density maps obtained from each MD trajectory in putative of E2 state. **f** The probabilities of POPA, POPS, POPI and PIP2 bound on the surface of hATP13A2 around the cytosolic cavity. The solid-colored histograms represent the original proportions (left) of the negatively charged phospholipids and the black slashed ones are the probabilities of lipids from simulations (right).

Supplementary Fig. 16

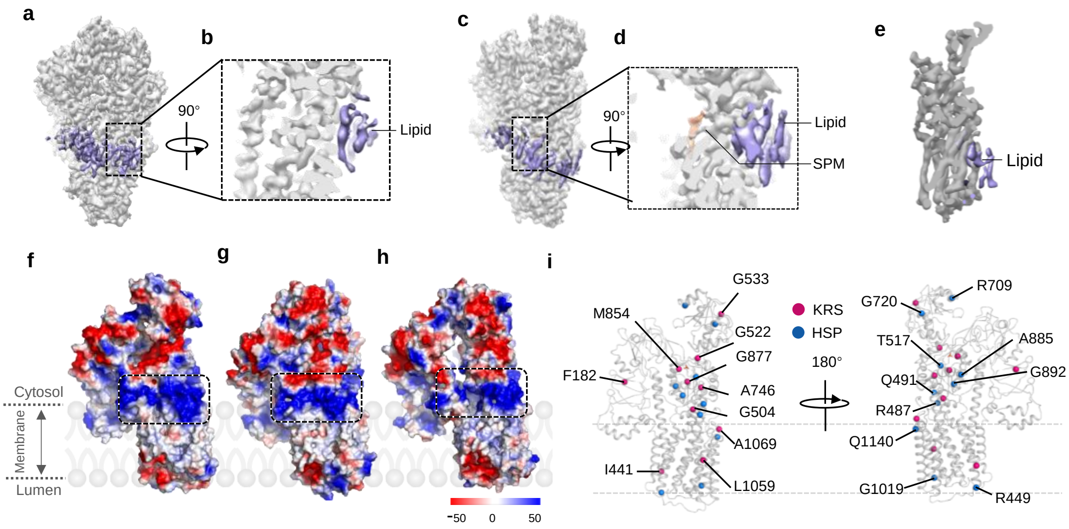

Supplementary Fig. 16 EM map analysis and disease-associated mutation of hATP13A2. **a, c, e** EM density of the lipid band bound to transmembrane domain at E1-ATP (a), E2-Pi (c) and putative E2 state (e), respectively. **b, d** The distribution of lipids in the transmembrane region at the E1-ATP state (b) and E2-Pi state (d), respectively. **f-h** Electrostatic potential molecular surface shows the large positive potential belt on the side adjacent to the cytoplasm of E1-ATP (f), E2-Pi (g), and putative E2 state (h), respectively. **i** Three-dimensional mapping of disease-associated missense mutations on the ATP13A2 structure. The residues of the disease-causing mutation site are rendered as red spheres (related to KRS) and blue spheres (related to hereditary spastic paraplegia (HSP)).

Supplementary Table 1 Cryo-EM data collection, refinement and validation statistic

	**E1-like**<br>**EMD-35385**<br>**PDB: 8IEL**	**Putative E2**<br>**EMD-35391**<br>**PDB: 8IER**	**E1P-ADP**<br>**EMD-35392**<br>**PDB: 8IES**	**Nominal E1P**<br>**EMD-35388**<br>**PDB: 8IEO**	**E2-Pi**<br>**EMD-35387**<br>**PDB: 8IEN**	**E1-ATP**<br>**EMD-35384**<br>**PDB: 8IEK**	**E2P**<br>**EMD-35386**<br>**PDB: 8IEM**
Microscope	Titan Kirios	Titan Kirios	Titan Kirios	Titan Kirios	Titan Kirios	Titan Kirios	Titan Kirios
Magnification	81,000×	64,000×	81,000×	81,000×	81,000×	81,000×	81,000×
Voltage (kV)	300	300	300	300	300	300	300
Camera	Gatan K3 Summit	Gatan K3 Summit	Gatan K3 Summit	Gatan K3 Summit	Gatan K3 Summit	Gatan K3 Summit	Gatan K3 Summit
Camera mode	Super-resolution	Super-resolution	Super-resolution	Super-resolution	Super-resolution	Super-resolution	Super-resolution
Electron exposure(e^{-}/Å^2)	50	50	50	50	50	50	50
Defocus range (μm)	1.2 to 1.8	1.2 to 1.8	1.2 to 1.8	1.2 to 1.8	1.2 to 1.8	1.2 to 1.8	1.2 to 1.8
Pixel size (Å)	1.095	1.08	1.095	1.095	1.08	1.095	1.095
Movies	15, 333	12, 419	1,427	4, 049	4, 233	2, 818	3, 608
Frames/movie	32	32	32	32	32	32	32
Symmetry imposed	C1	C1	C1	C1	C1	C1	C1
Initial particle projections (no.)	4,422,329	4,888,946	1,126,131	2,743,122	1,917,434	3,094,372	4,948,958
Final particle projections(no.)	242,367	394,767	151,804	266,168	290,927	325,325	242,437
Map resolution (Å)	5.65	4.87	3.73	3.78	3.25	3.2	3.35
FSC threshold	0.143	0.143	0.143	0.143	0.143	0.143	0.143
Map resolution range (Å)	3 to 10	2 to 10	2 to 8	2 to 8	2 to 8	2 to 8	2 to 8
Initial model used	De novo	De novo	De novo	De novo	De novo	De novo	De novo
Software	cryoSPARC	cryoSPARC	cryoSPARC	cryoSPARC	cryoSPARC	cryoSPARC	cryoSPARC
Model resolution (Å)	5.65	4.87	3.73	3.78	3.25	3.2	3.35
FSC threshold	0.143	0.143	0.143	0.143	0.143	0.143	0.143
Map sharpening B factor (Å^2)	530.3	354.3	155.4	148.1	106.2	131.1	149.1
Model composition							
Non-hydrogen atoms	7933	7534	7965	8026	7739	8045	7855
Protein residues	1025	968	1026	1031	992	1036	1011
Ligand	0	1	3	3	4	2	3
R.m.s. deviations							
Bond lengths (Å)	0.002	0.022	0.002	0.008	0.014	0.005	0.010
Bond angles (°)	0.698	0.693	0.550	0.849	1.175	0.825	1.301
MolProbity score	1.89	1.93	1.68	1.77	1.69	1.72	1.77
Clashscore	8.98	9.03	5.19	7.72	6.33	6.06	7.00
Ramachandran plot							
Favored (%)	93.67	93.05	94.00	96.16	95.09	94.24	94.38
Allowed (%)	6.33	6.95	6.00	4.84	4.91	5.76	5.32

Supplementary Table 2 The composition of asymmetric lipid bilayer in the simulation.

Lumenal leaflet		Cytosolic leaflet	
Lipid type	Lipid number	Lipid type	Lipid number
CHOL	145 (38.8%)	CHOL	54 (15.2%)
POPC	173 (46.2%)	POPC	70 (19.8%)
POSM	56 (15.0%)	POPE	113 (32.0%)
		POPA	81 (23.0%)
		POPS	10 (3.0%)
		POPI	12 (3.5%)
		PIP2	12 (3.5%)

Supplementary Table 3 Published structural information for ATP13A2 and homologous proteins in different intermediate states. Related to Supplementary Fig. 10.

PDB ID	State	EM Map	Mutation(s)	Expression System	Reference
**7OP5**	E2P*	EMD-13013	No	Saccharomyces cerevisiae	Li P et al., 2021
**7OP8**	$E2P^{inhibit}$	EMD-13014	No	Saccharomyces cerevisiae	
**7OP1**	$E2Pi^{AlF/SPM}$	EMD-13011	No	Saccharomyces cerevisiae	
**7OP3**	$E2Pi^{SPM}$	EMD-13012	No	Saccharomyces cerevisiae	
**7N70**	E2P-like	EMD-24214	No	Spodoptera frugiperda	Sim S I et al., 2021
**7N72**	E2-Pi-like	EMD-24217	No	Spodoptera frugiperda	
**7N78**	E2-Pi, post-hydrolysis	EMD- 24223	No	Spodoptera frugiperda	
**7N73**	E1P-ADP like	EMD-24218	No	Spodoptera frugiperda	
**7N74**	E1-ATP	EMD-24219	D508N	Spodoptera frugiperda	
**7N75**	E1-apo	EMD-24220	D458N/D962N	Spodoptera frugiperda	
**7N76**	E1-apo	EMD-24221	D458N/D962N	Spodoptera frugiperda	
**7N77**	E1P-like	EMD-24222	D458N/D962N	Spodoptera frugiperda	
**7M5V**	E1-AMPPNP	EMD-23683	No	Homo sapiens	Tillinghast J., 2021
**7M5X**	SPM-E2-BeF_3^-	EMD-23684	No	Homo sapiens	
**7M5Y**	SPM-E2-MgF_4^{2-}	EMD-23685	No	Homo sapiens	
**7VPJ**	E1P-ADP	EMD-32067	No	Homo sapiens	Tomita A et al., 2021
**7VPI**	E1ATP	EMD-32066	No	Homo sapiens	
**7VPL**	E2Pi(SPM)	EMD-32069	No	Homo sapiens	
**7VPK**	E2P(SPM)	EMD-32068	No	Homo sapiens	
**7FJM**	E1	EMD-31623	No	Homo sapiens	Chen X D et al., 2021
**7FJP**	E1P-ADP	EMD-31626	No	Homo sapiens	
**7FJQ**	E2-Pi	EMD-31627	No	Homo sapiens	

Supplementary Table 4. Primers for cloning of ATP13A2 and point mutations in this study

Prime name	Prime sequence
A13-F	5'- TTCGGATCCGCCACCATGAGCGCAGACAGCAGCC -3'
A13-R	5'- TCCACCCCCCTCGAGCCTCAGGGGGCCGGCGGGC -3'
Y240A-F	5'- AACCCCgccTATGGGTTCCAGGCCTTCAGCAT -3'
Y240A-R	5'- AACCCATAggcGGGGTTCAGTGCCTCGTCCAC -3'
F428A-F	5'- AACACAGCATGAAGgccGTGGCTGCCCTCTCTGTCCT -3'
F428A-R	5'- ggcCTTCATGCTGTGTTTATAGAACTTGAAGT -3'
F934A-F	5'- AGCGTCgccAAGTACATGGCTCTGTACAGCCTG -3'
F934A-R	5'- ATGTACTTggcGACGCTGAACGAAGTGTCAAGG -3'
F419A-F	5'- CATCAACgccAAGTTCTATAAACACAGCATGAAGTTTG -3'
F419A-R	5'- AGAACTTggcGTTGATGGGCCGGGGGTGCAAG -3'
L432A-F	5'- TTGTGGCTGCCgccTCTGTCCTGGCTCTCCTCGG -3'
L432A-R	5'- AGAggcGGCAGCCACAAACTTCATGCTGTGTT -3'
E235A-F	5'- GTGGACgccGCACTGAACCCCTACTATGGGTT -3'
E235A-R	5'- TTCAGTGCggcGTCCACCAGCAGCTGGGGGTA -3'
N238A-F	5'- CACTGgccCCCTACTATGGGTTCCAGGCCTTC -3'
N238A-R	5'- ATAGTAGGGggcCAGTGCCTCGTCCACCAGCA -3'
K277A-F	5'- CTGTACgccACCAGAAAGCAAAGCCAGACTCT -3'
K277A-R	5'- TTTCTGGTggcGTACAGCGACAGGCAGATGGA -3'
K241A-F	5'- AACCCCTACgccGGGTTCCAGGCCTTCAGCAT -3'
K241A-R	5'- AACCCggcGTAGGGGTTCAGTGCCTCGTCCAC -3'
S270A-F	5'- TTCCTCCATCgccATCTGCCTGTCGCTGTACAAGA -3'
S270A-R	5'- AGATggcGATGGAGGAAATGAGGAAGATGCAC -3'
D513N-F	5'- GTGTTTCaacAAGACGGGCACCCTCACTGAGG -3'
D513N-R	5'- CCGTCTTgttGAAACACACCAGCTGCAGCTTG -3'
