# Supplementary material for: Conformational cycle of human polyamine transporter ATP13A2
Source: Nat Commun. 2023 Apr 8;14:1978. doi: 10.1038/s41467-023-37741-0 (PMC10082790; doi:10.1038/s41467-023-37741-0)
Supplement: Supplementary file 3 — Description of Additional Supplementary Files [file 41467_2023_37741_MOESM3_ESM.pdf]

File name: Supplementary Data 1

Description: A dataset for cross-linking mass spectrometry of wild-type ATP13A2 (a) and Y240A mutant (b) using a BS^3 crosslinker, respectively. Cross-linked search results are divided into cross-linked, link-linked, mono-linked and SPM-ATP13A2(WT/Y240) according to the form of the cross-linked, and the data are placed separately in the sheet.

File name: Supplementary Movie 1

Description: Potential releasing pathways connecting the releasing site and cytosolic side. The all-atom MD simulation shows that the highly hydrated channels are shown in dark blue.

File name: Supplementary Movie 2

Description: A proposed model of hATP13A2 transporting polyamines.

Atomic models of hATP13A2 proteins shown as ribbons according to the default color code. The membrane structure is marked in pink. Schematic diagram of Post-Albers cycle for ATP13A2 is shown in the lower left corner, and the yellow dots indicate a conformational change of hATP13A2 corresponding to Post-Albers cycle during substrate transport. The lipid tails is green and the head is red. ATP and SPM are shown as spherical models in magenta and blue, respectively.
