# Supplementary material for: Conformational cycle of human polyamine transporter ATP13A2
Source: Nat Commun. 2023 Apr 8;14:1978. doi: 10.1038/s41467-023-37741-0 (PMC10082790; doi:10.1038/s41467-023-37741-0)
Supplement: Supplementary file 7 — Reporting Summary [file 41467_2023_37741_MOESM7_ESM.pdf]

nature portfolio

Corresponding author(s): Zhongmin Liu

Last updated by author(s): Mar 12, 2023

Reporting Summary

Nature Portfolio wishes to improve the reproducibility of the work that we publish. This form provides structure for consistency and transparency in reporting. For further information on Nature Portfolio policies, see our Editorial Policies and the Editorial Policy Checklist.

Statistics

For all statistical analyses, confirm that the following items are present in the figure legend, table legend, main text, or Methods section.

n/a	Confirmed	
☐	☒	The exact sample size (*n*) for each experimental group/condition, given as a discrete number and unit of measurement
☐	☒	A statement on whether measurements were taken from distinct samples or whether the same sample was measured repeatedly
☐	☒	The statistical test(s) used AND whether they are one- or two-sided<br>*Only common tests should be described solely by name; describe more complex techniques in the Methods section.*
☒	☐	A description of all covariates tested
☒	☐	A description of any assumptions or corrections, such as tests of normality and adjustment for multiple comparisons
☐	☒	A full description of the statistical parameters including central tendency (e.g. means) or other basic estimates (e.g. regression coefficient) AND variation (e.g. standard deviation) or associated estimates of uncertainty (e.g. confidence intervals)
☐	☒	For null hypothesis testing, the test statistic (e.g. *F*, *t*, *r*) with confidence intervals, effect sizes, degrees of freedom and *P* value noted<br>*Give P values as exact values whenever suitable.*
☒	☐	For Bayesian analysis, information on the choice of priors and Markov chain Monte Carlo settings
☒	☐	For hierarchical and complex designs, identification of the appropriate level for tests and full reporting of outcomes
☒	☐	Estimates of effect sizes (e.g. Cohen's *d*, Pearson's *r*), indicating how they were calculated

Our web collection on statistics for biologists contains articles on many of the points above.

Software and code

Policy information about availability of computer code

Data collection	cryo-EM data were collected with Serial-EM V3.7.0 and EPU V2.8.1
Data analysis	MotionCor2 v1.4.2, cryoSPARC v3.2, CTFFIND4, Phyre2 V 2.0, Coot v0.8.9, Phenix v1.13, Pymol v2.2, UCSF ChimeraX v1.3, Martini 2.2, NAMD version 2.14, CHARMM36m, CHARMM-GUI web interface, CHARMM General Force Field, Gromacs 2019.6, 2020.3 or 2020.6, martinize.py v2.4, GROmaps, pLabel v2.4.05, GraphPad Prism v 9.0.0

For manuscripts utilizing custom algorithms or software that are central to the research but not yet described in published literature, software must be made available to editors and reviewers. We strongly encourage code deposition in a community repository (e.g. GitHub). See the Nature Portfolio guidelines for submitting code & software for further information.

Data

Policy information about availability of data

All manuscripts must include a data availability statement. This statement should provide the following information, where applicable:

- Accession codes, unique identifiers, or web links for publicly available datasets
- A description of any restrictions on data availability
- For clinical datasets or third party data, please ensure that the statement adheres to our policy

The cryo-EM maps and the coordinates in this study have been deposited in the Electron Microscopy Data Bank (EMDB) and Protein Data Bank (PDB) under accession codes: EMD-35384 and 8IEK for E1-ATP state; EMD-35385 and 8IEL for E1-like state; EMD-35392 and 8IES for E1P-ADP state; EMD-35386 and 8IEM for

E2P state; EMD-35387 and 8IEN for E2-Pi state; EMD-35388 and 8IEO for nominal E1P state; EMD-35391 and 8IER for putative of E2 state. Mass spectrometry proteomics data have been deposited to ProteomeXchange Consortium (http://proteomecentral.proteomexchange.org) via the PRIDE partner repository with the dataset identifier PXD037493.

Human research participants

Policy information about studies involving human research participants and Sex and Gender in Research.

Reporting on sex and gender	none
Population characteristics	none
Recruitment	none
Ethics oversight	none

Note that full information on the approval of the study protocol must also be provided in the manuscript.

Field-specific reporting

Please select the one below that is the best fit for your research. If you are not sure, read the appropriate sections before making your selection.

☒ Life sciences ☐ Behavioural & social sciences ☐ Ecological, evolutionary & environmental sciences

For a reference copy of the document with all sections, see nature.com/documents/nr-reporting-summary-flat.pdf

Life sciences study design

All studies must disclose on these points even when the disclosure is negative.

Sample size	For each ATP13A2 complex, one cryo-EM dataset comprising several thousand micrographs were collected (shown in Supplementary Table 1). The number of no specific statistical methods were used to determine sample size as it was not generally applicable to our study. For electron microscopy data, sample size was determined by the availability of areas to image on grids.
Data exclusions	No data were excluded
Replication	The number of the replication is stated in the Figure Legends.
Randomization	Randomization was not generrally used in this study. No human or animal subjects were used in the study.
Blinding	Blinding was not necessary for this studies because no subjective allocation was involved.

Reporting for specific materials, systems and methods

We require information from authors about some types of materials, experimental systems and methods used in many studies. Here, indicate whether each material, system or method listed is relevant to your study. If you are not sure if a list item applies to your research, read the appropriate section before selecting a response.

Materials & experimental systems

n/a	Involved in the study	
☒	☐	Antibodies
☐	☒	Eukaryotic cell lines
☒	☐	Palaeontology and archaeology
☒	☐	Animals and other organisms
☒	☐	Clinical data
☒	☐	Dual use research of concern

Methods

n/a	Involved in the study	
☒	☐	ChIP-seq
☒	☐	Flow cytometry
☒	☐	MRI-based neuroimaging

Eukaryotic cell lines

Policy information about cell lines and Sex and Gender in Research

Cell line source(s)	HEK293F (Thermo Fisher Scientific)

Authentication	No
Mycoplasma contamination	The cell lines were not tested for Mycoplasma contamination.
Commonly misidentified lines (See ICLAC register)	No
